# Pharmacokinetic-pharmacodynamic relationships of pour-on administered eprinomectin in nematode-infected lactating female and male castrated dairy breed goats

**DOI:** 10.1007/s00436-022-07483-x

**Published:** 2022-03-12

**Authors:** Xiuqing Gao, Valerie Kvaternick, Steffen Rehbein, Dietmar Hamel

**Affiliations:** 1 Formerly of Boehringer Ingelheim Animal Health USA, Inc., DSD, 631 Route 1 South, North Brunswick, NJ 08902 USA; 2 Boehringer Ingelheim Animal Health USA, Inc., DSD, 631 Route 1 South, North Brunswick, NJ 08902 USA; 3 Boehringer Ingelheim Vetmedica GmbH, Kathrinenhof Research Center, Walchenseestraße 8-12, 83101 Rohrdorf, Germany

**Keywords:** Goat, Eprinomectin, Lactating, Pharmacokinetic-pharmacodynamic (PK-PD)

## Abstract

Eprinomectin (EPM), a macrocyclic lactone with low excretion in milk and high efficacy against endoparasites and ectoparasites, is widely used in veterinary medicine. In this paper, EPM pharmacokinetics and anthelmintic efficacy previously established in one study with lactating female goats and three studies with male castrated growing dairy breed goats (all with induced mixed adult gastrointestinal nematode parasitism and treated with a single 1-mg/kg pour-on administration of EPM) were retrospectively evaluated using pharmacokinetic-pharmacodynamic (PK-PD) modeling. The PK-PD analyses between EPM exposure (*C*_max_ and AUC_last_) and anthelmintic response (percent efficacy) were performed for lactating female goats only and pooled lactating female and male castrated goats. The *C*_max_ and AUC_last_ showed no significant difference between lactating female goats and combined male castrated goats. PK-PD modeling demonstrated *Trichostrongylus colubriformis*, a small-intestine nematode, as a suitable indicator of the EPM nematocidal efficacy. The EC_90_ values obtained by modeling *C*_max_ vs *T. colubriformis* were 3.50 and 2.43 ng/mL for lactating female goats only and pooled lactating female and male castrated goats, respectively. The values of AUC_last_ needed for 90% efficacy of *T. colubriformis* were 25.4 and 21.1 day*ng/mL for lactating female goats only and pooled lactating female and male castrated goats, respectively. Overall, the predicted pharmacological response against *T. colubriformis* is similar for lactating goats only and pooled lactating female and male castrated goats and correlates with observed efficacy. In conclusion, a dosage of 1-mg/kg EPM as a pour-on is sufficient to ensure efficacy against common nematodes in both lactating female and male castrated goats.

## Introduction

Eprinomectin (EPM) is a macrocyclic lactone characterized by a broad spectrum of endoparasiticidal and ectoparasiticidal activity and low milk partitioning (Shoop et al. 1996a). These favorable properties allow for its use as an antiparasitic treatment in veterinary medicine, especially as a topical EPM 5-mg/mL product for pour-on administration to cattle, sheep, and goats with 0-h milk withdrawal (Shoop et al. 1996b; Shoop and Soll 2002; Hamel et al. 2017, 2021).

It is accepted that goats need EPM administered as a pour-on at a higher dosage compared to cattle (1-mg/kg bodyweight vs 0.5-mg/kg bodyweight, respectively) (Chartier and Pors 2004; Cringoli et al. 2004; Hamel et al. 2015, 2021; Rehbein et al. 2014). The dosage for goats is, nevertheless, a matter of debate, mainly focusing on female dairy goats as lactation is considered a physiological covariate which may alter the pharmacokinetics (PK), and thus, the pharmacological response or anthelmintic efficacy of EPM (Dupuy et al. 2001; Lespine et al. 2012; Rostang et al. 2020). Apart from an earlier PK (only) study in lactating female dairy goats (Dupuy et al. 2001), only one publication presents PK and efficacy data established in the same study in lactating female dairy goats that confirms the efficacy of a single pour-on EPM treatment at 1 mg/kg against induced infections with a range of important gastrointestinal and respiratory nematodes of goats (Hamel et al. 2021).

To further corroborate the marketed dosage in goats of pour-on EPM (1-mg/kg bodyweight), results of a series of four combined efficacy and PK studies in lactating female and male castrated growing dairy breed goats (Rehbein et al. 2014; Hamel et al. 2015, 2021) were retrospectively analyzed to investigate the PK-PD relationship of the pour-on EPM treatment, as an in-depth understanding of the anthelmintic properties of formulated products is a prerequisite for their effective use (Vercruysse et al. 2018).

## Methods

For this investigation, PK-PD relevant data were extracted from four previously published studies which included induced mixed adult gastrointestinal strongylid nematode (*Haemonchus contortus*, *Teladorsagia circumcincta*, *Trichostrongylus axei*, *Trichostrongylus colubriformis*, *Cooperia curticei*, *Nematodirus battus*, *Nematodirus spathiger*, and/or *Oesophagostomum venulosum*) infected lactating female (Hamel et al. 2021 — Study 1) or male castrated growing dairy breed goats (Rehbein et al. 2014 — Study 2; Hamel et al. 2015 — Studies 3 and 4). These controlled anthelmintic efficacy studies followed VICH guidelines (Vercruysse et al. 2001), had an essentially identical study design, and supported the registration in goats in Europe of the 5-mg/mL EPM topical product (EPRINEX® Multi, Boehringer Ingelheim) administered as a pour-on at 1-mg/kg bodyweight. In short, animals in each study were allocated into two groups (control [untreated] or EPM-treated) of equal size (*n* = 10 for Study 1; *n* = 8 each for Studies 2, 3, and 4) and 14 days after pour-on administration of the commercial 5-mg/mL EPM topical product at 1 mg/kg to one group, all animals were necropsied to determine the treatment efficacy based on adult nematode counts. In addition, blood samples were collected in intervals up to 14 days after EPM treatment and plasma was analyzed to determine the EPM plasma concentration profile.

Plasma concentrations in the four studies were previously analyzed by non-compartmental analysis using WinNonlin® Software (version 5.0 or higher). Presently, a non-parametric ANOVA was used to analyze differences of established PK parameters (*C*_max_, AUC_last_) between lactating female goats (Study 1) and combined male castrated goats (Studies 2, 3, and 4). The significance level was set at *α* = 0.05.

Individual percent efficacies per nematode species based on adult nematode counts established 14 days following pour-on EPM treatment at 1-mg/kg bodyweight of the goats were calculated as follows: (1 − [count for EPM-treated goats] / [geometric mean count for the untreated controls]) × 100.

A sigmoid *E*_max_ model (model 105, where effect *C* = 0 at 0, *C* = infinity at *E*_max_) from Phoenix WinNonlin® (build 8.1.0.3530) was used for this current PK-PD analysis using lactating female goat data only (Study 1) and a mixed population of growing male castrated and lactating female goats data (Studies 1, 2, 3, and 4) based on the assumption of a direct relationship of plasma exposure (*C*_max_ and AUC_last_) to pharmacological response (anthelmintic percent efficacy). The sigmoid *E*_max_ model used was

$$\mathrm E=\frac{\mathrm{Emax}\times\mathrm C^{\mathrm{Gamma}}}{\mathrm C^{\mathrm{Gamma}}+{\mathrm{EC}50}^{\mathrm{Gamma}}}$$

where Gamma is the the Hill coefficient which describes the slope of the exposure effect curve, *C* is the exposure (*C*_max_ and AUC_last_), *E* is the anthelmintic response, *E*_max_ is the maximum anthelmintic efficacy, and EC_50_ is the exposure producing 50% of *E*_max_. An exposure producing 90% of *E*_max_ (EC_90_) was calculated using the following equation:

$$\mathrm{EC}90=\mathrm{EC}50 \times {\left[\frac{90}{100-90}\right]}^{\frac{1}{\mathrm{Gamma}}}$$

## Results

The pharmacokinetic parameters *C*_max_ and AUC_last_ were not significantly different between lactating female goats only and combined male castrated goats (*p* > 0.05).

PK-PD analysis of EPM activity was performed by building an exposure–response relationship between EPM exposure data (*C*_max_, AUC_last_) from lactating female goats only (Study 1) or pooled lactating female and male castrated goats (Studies 1, 2, 3, and 4 combined). PK-PD analysis for male castrated goats only was not performed due to the lack of a sufficient number of individuals showing < 90% anthelmintic efficacy. For lactating female goats only and pooled lactating female and male castrated goats, only the small-intestine nematode *T. colubriformis* data could be described by the PK-PD model. The sigmoid *E*_max_ model was not appropriate to describe the other nematode species data sets as all values were close to *E*_max_.

**Fig. 1:**
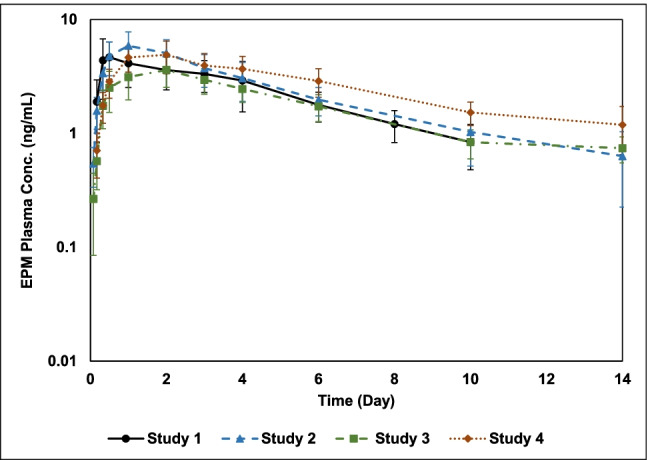
Mean (± SD) plasma EPM concentration time profiles following a single EPM pour-on administration at 1 mg/kg to lactating female (Study 1; *n* = 10) and male castrated growing (Studies 2, 3, and 4; *n* = 8 per study) dairy breed goats (log-linear scale)

The observed PK and efficacy data for *T. colubriformis* of the four studies (including pooled studies data) are shown in Table 1, and EPM concentration vs time profiles are presented in Fig. 1 (Study 1 up to day 12; Studies 2, 3, and 4 up to day 14).

**Table 1:** Summary of PK parameters and efficacy against *Trichostrongylus colubriformis* of EPM following a single pour-on administration of a topical 5-mg/mL EPM product at 1 mg/kg to lactating female (Study 1) and male castrated growing (Studies 2, 3, and 4) dairy breed goats

Study/studies (number of EPM-treated goats)	PK parameters, mean (± SD)^1^		Efficacy (%) against *Trichostrongylus colubriformis*	
	*C*_max_ (ng/mL)	AUC_last_ (day*ng/mL)	Per study^2^	Pooled over studies^3^
1 (*n* = 10)	5.35 (± 2.27)	23.8 (± 9.73)	97.0	Not applicable
2, 3, 4 (*n* = 24)	4.94 (± 1.73)	30.2 (± 9.26)	99.6, 98.7, 96.5	98.3
1, 2, 3, 4 (*n* = 34)	5.06 (± 1.88)	28.3 (± 9.72)	97.0, 99.6, 98.7, 96.5	97.6

^1^Mean and SD of the PK parameters were calculated using individual goat’s values with three significant numbers

^2^Efficacy (%), per study = 100 × [(*C* − *T*)/*C*], where *C* is the geometric mean of the individual *T. colubriformis* counts of the controls and *T* is the geometric mean of the individual *T. colubriformis* counts of the EPM-treated goats

^3^Pooled efficacy (%) was calculated as the arithmetic mean of the individual study percentage efficacies

**Fig. 2:**
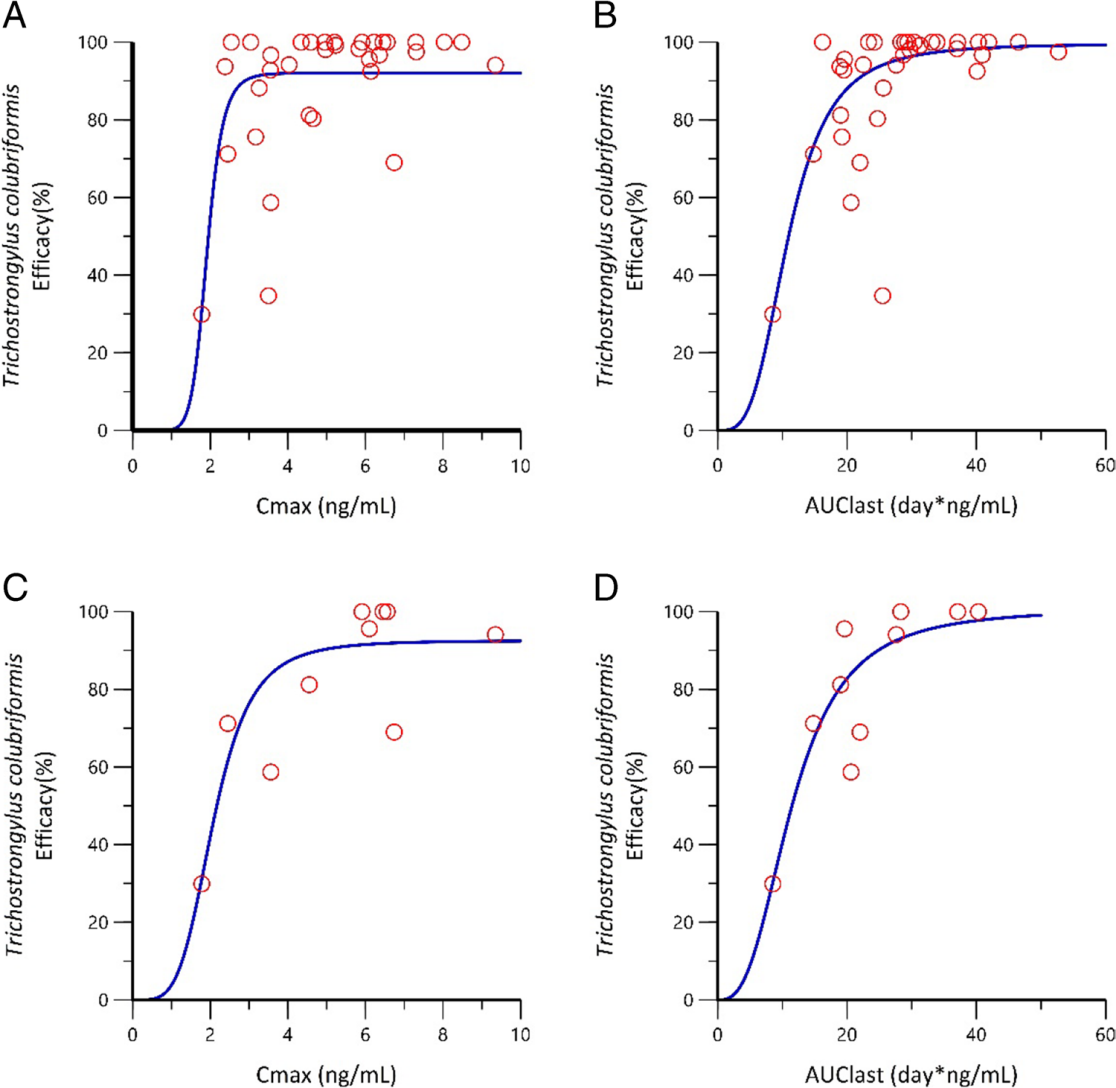
Sigmoid *E*_max_ model fit of EPM exposure (EPM *C*_max_ and EPM AUC_last_) vs efficacy (%) against *Trichostrongylus colubriformis* in pooled lactating female and male castrated growing dairy breed goats (Studies 1, 2, 3, and 4; *n* = 34): **A** and **B** lactating female dairy breed goats only (Study 1; *n* = 10) and **C** and **D** a single EPM pour-on administration at 1 mg/kg (red circles = observed individual experimental data, blue line = fit to sigmoid curve)

Results of the PK-PD modeling for EPM treatment vs *T. colubriformis* efficacy are summarized in Table 2 and modeled exposure response curves are presented in Fig. 2. The *E*_max_ was > 92% for *T. colubriformis* when response relationships were modeled against *C*_max_ and AUC_last_. The EC_50_ values obtained by modeling *C*_max_ vs response for *T. colubriformis* using pooled lactating female and male castrated goat data and lactating female only data were 1.92 and 2.09 ng/mL, respectively. The EC_90_ values were 2.43 and 3.50 ng/mL, respectively, for the pooled data and the lactating female only data. The AUC_last_ values estimated for 50% efficacy of *T. colubriformis* using pooled lactating female and male castrated goat data and lactating female only data were 10.9 and 11.5 day*ng/mL, respectively. The AUC_last_ values estimated for 90% efficacy of *T. colubriformis* were 21.1 and 25.4 day*ng/mL, respectively, for the pooled data and the lactating female only data. Taking the EC_90_ as the predictor of acceptable efficacy, the observed (calculated) EPM exposure (*C*_max_ and AUC_last_) from pooled lactating female and male castrated goats met or exceeded the predicted EC_90_ values. Observed results showed ≥ 96.5% efficacy of the EPM treatment. For lactating female goats only, *C*_max_ met or exceeded the predicted EC_90_ and AUC_last_ was slightly less than the predicted 90% efficacy level. The observed results showed 97.0% efficacy of the EPM treatment in lactating female goats.

**Table 2:** PK-PD parameters (EPM *C*_max_ and EPM AUC_last_ vs efficacy relationships) of EPM treatment against *Trichostrongylus colubriformis* following a single EPM pour-on administration of a topical 5-mg/mL EPM product at 1 mg/kg to a mixed population of growing male castrated and lactating female dairy breed goats (Studies 1, 2, 3, and 4; *n* = 34) and lactating female dairy breed goats only (Study 1; *n* = 10)

PK-PD parameter	Goat population analyzed	*C*_max_ (ng/mL)	AUC_last_ (day*ng/mL)
*E*_max_ (%)	Pooled lactating female + male castrated growing dairy breed goats	92.1 ± 2.84	99.6 ± 4.94
	Lactating female dairy breed goats	92.6 ± 6.95	101 ± 13.1
EC_50_/50% efficacy	Pooled lactating female + male castrated growing dairy breed goats	1.92 ± 0.09	10.9 ± 0.83
	Lactating female dairy breed goats	2.09 ± 0.17	11.5 ± 1.77
EC_90_/90% efficacy	Pooled lactating female + male castrated growing dairy breed goats	2.43 ± 0.11	21.1 ± 1.60
	Lactating female dairy breed goats ats	3.50 ± 0.29	25.4 ± 3.89
Gamma	Pooled lactating female + male castrated growing dairy breed goats	9.19 ± 3.65	3.35 ± 0.72
	Lactating female dairy breed goats	4.26 ± 1.74	2.78 ± 0.90

## Discussion

The objective of this paper was to assess the dose–response of pour-on EPM administered at 1-mg/kg bodyweight against infections with apparently macrocyclic lactone-sensitive gastrointestinal strongylid nematodes in goats. PK-PD analysis is helpful to establish safe and effective dosage regimens of drugs for animals as suboptimal dosing is considered one of the major factors contributing to emergence of resistant pathogens (Toutain 2002; Lanusse et al. 2018).

The observed PK results in the four studies retrospectively evaluated indicate that EPM exposure in lactating female goats was comparable to male castrated goats (Fig. 1). Mean PK parameters for both *C*_max_ and AUC_last_ for lactating female goats were comparable to male castrated goats (Table 1). Although to be confirmed in a PK-PD study in female non-lactating dairy breed goats administered pour-on EPM at 1-mg/kg bodyweight, the outcome of the combined analysis of the PK-PD data presented here may indicate that lactating status does not alter the exposure of EPM in dairy goats. In addition to lactation, other factors such as age, bodyweight, body condition, breed, milk production, health status, infection, and geographical conditions may influence the PK profile of drugs (Lespine et al. 2012).

The PK-PD modeling presented here demonstrated *T. colubriformis* as a suitable nematode species for modeling based on lactating female goat data and mixed populations of growing male castrate and lactating female goat data. Due to maximal/near-maximal efficacy against the other species of strongylid nematodes included in the four combined PK-efficacy studies evaluated, their data were not amenable to the sigmoid *E*_max_ model. Based on observed and modeled EPM exposure, predicted EC_90_ values were reached for *C*_max_ in lactating female goats and mixed populations of growing male castrated and lactating female goats, and for AUC_last_ in mixed populations of growing male castrated and lactating female goats. This was in line with the (calculated) pooled efficacy of 97.6%. In lactating female goats, the observed AUC_last_ was similar to the predicted value (23.8 vs 25.4 day*ng/mL) and correlated with the observed EPM efficacy of 97.0%, which indicates that minimum nematocidal drug concentrations were maintained over a sufficient time to achieve the required efficacy as discussed previously (Hamel et al. 2021).

Among the strongylid nematodes parasitizing the gastrointestinal tract of domestic ruminants, the species dwelling in the small intestine are the least sensitive to macrocyclic lactones (Egerton et al. 1981; Shoop et al. 1996a). Although not investigated to the extent as done with cattle-specific nematodes, which identified the small-intestine nematodes of the genus *Cooperia* as dosage-limiting or dosage-discriminating nematodes for macrocyclic lactones (Egerton et al. 1981; Shoop et al. 1996b, 2001; Vercruysse and Rew 2002), there have been titration studies reported which included *T. colubriformis*, a parasite of the small intestine too. The studies testing parenterally administered compounds of the macrocyclic lactone class showed this parasite as least responsive or less responsive to treatment or exhibiting dosage discriminating properties (Egerton et al. 1981; Shoop et al. 1996b). Thus, historical information as well as recently observed efficacy data of EPM against *T. colubriformis* show that this species can be used as a predictor of efficacy of other strongylid nematodes of the gastrointestinal tract of domestic ruminants.

In summary, the PK-PD analysis of EPM in goats confirms that the dosage of 1 mg/kg EPM administered as a pour-on is sufficient to ensure efficacy against common gastrointestinal strongylid nematodes in both lactating female and male castrated dairy breed goats. A PK-PD relationship was established and indicates that *C*_max_ and AUC_last_ are good predictors of EPM efficacy against *T. colubriformis*, which can serve as representative for other gastrointestinal strongylid nematodes.

## Declarations

### Disclaimer

EPRINEX® is a registered trademark of Boehringer Ingelheim. All other marks are property of their respective owners. This document is provided for scientific purposes only. Any reference to a brand or trademark herein is for informational purposes only and is not intended for a commercial purpose or to dilute the rights of the respective owner(s) of the brand(s) or trademark(s).
